# Reimagining Indigenous healthcare through a readiness to practice lens: A quantitative content analysis of the empirical literature

**DOI:** 10.17269/s41997-024-00989-0

**Published:** 2025-03-31

**Authors:** Tyara Marchand, Adam Murry, Devin Proulx, K. Alix Hayden, Lynden Crowshoe

**Affiliations:** 1 Cumming School of Medicine, University of Calgary, Calgary, AB Canada; 2 Department of Psychology, University of Calgary, Calgary, AB Canada; 3 College of Medicine, University of Saskatchewan, Saskatoon, SK Canada; 4 University of Calgary Library, Calgary, AB Canada; 5 Department of Family Medicine, Cumming School of Medicine, University of Calgary, Calgary, AB Canada

**Keywords:** Indigenous health services, Patient-centred care, Readiness to practice, Clinical competence, Services de santé autochtones, Soins axés sur le patient, Préparation à la pratique, Compétence clinique

## Abstract

**Objectives:**

The concept of “readiness to practice” has not been clearly delineated within an Indigenous health context. This systematic review occurred on a multi-database survey of published primary literature. The primary objective of this review was to determine what it takes for clinicians to be ready to practice with Indigenous populations.

**Methods:**

This review identified articles published in the last 20 years within Canada, the United States, New Zealand, and Australia. The databases that were searched included CINAHL, Medline (via Ovid), Embase (via Ovid), Scopus, and Web of Science, with an additional hand search of references from relevant articles. This search took place from January to May 2022, with subsequent analysis from May to September 2022.

**Results:**

Primary studies were coded using quantitative content analysis procedures and quantified codes were subjected to exploratory factor analyses. Four factors described a competent clinician across studies, including a relational disposition, decolonized practice, cultural immersion, and Indigenous professional support.

**Conclusion:**

This sphere of literature is relatively novel and there do not appear to be many individuals directly commenting on attributes needed to be prepared to work with Indigenous communities. There exist potential gaps in knowledge that could be addressed by conversations with Indigenous stakeholders and implementation of health education programs that focus on developing Indigenous-specific competencies.

## Résumé

**Objectifs:**

Le concept de «prêt à pratiquer» n’est pas clairement élaboré dans le contexte de la santé autochtone. Une enquête a été menée sur plusieurs bases de données de littérature primaire publiée pour effectuer cet examen systématique. Cet examen visait principalement à déterminer ce qui est nécessaire pour que les cliniciens soient prêts à pratiquer la médecine avec des personnes autochtones.

**Méthodes:**

Cet examen a repéré des articles qui ont été publiés au Canada, aux États-Unis, en Nouvelle-Zélande et en Australie au cours des 20 dernières années. Les bases de données sélectionnées pour la recherche comprennent le CINAHL, Medline (via Ovid), Embase (via Ovid), Scopus et Web of Science, avec une recherche manuelle pour d’autres références d’articles pertinents. La recherche a été réalisée entre janvier et mai 2022, et l’analyse a été effectuée entre mai et septembre 2022.

**Résultats:**

Les études primaires ont été codifiées par des procédures d’analyse de contenu quantitative, et les codes quantifiés ont été soumis à une analyse factorielle exploratoire. Quatre facteurs décrient ce qui rend un clinicien compétent à travers les études, à savoir sa disposition relationnelle, sa pratique décolonisée, son immersion culturelle et son soutien professionnel autochtone.

**Conclusion:**

Ce domaine est récent, avec peu de commentaires qui mettent en avant les caractéristiques pour être prêt à travailler en collaboration avec des communautés autochtones. Il est possible de discuter des manques de connaissances avec des parties prenantes autochtones, en plus des programmes d’éducation de santé, afin de développer des compétences spécifiques pour le service aux peuples autochtones.

## Introduction

Throughout the developed world, Indigenous peoples continue to have increased health and wellness disparities as compared with their non-Indigenous counterparts. According to the United Nations, Indigenous peoples have increased rates of malnutrition, cardiovascular disease, heightened maternal and infant mortality, HIV/AIDS, and other infectious diseases, including COVID-19 (Axelsson et al., 2016; Clark et al., 2021). Indigenous peoples’ health is complicated in colonized countries where settlers both introduced diseases and enacted policies with negative health outcomes. Settler colonialism has differentially impacted Canada’s Indigenous peoples and continues to do so, specifically through the intergenerational trauma left behind from former government initiatives, including residential schools, the 60s Scoop, and Indian hospitals. This is echoed with the 2015 Truth and Reconciliation’s Calls to Action, where they call upon the federal government to acknowledge that the current state of health with Canada’s Indigenous peoples is directly caused by past federal strategies (Axelsson et al., 2016; HealthCareCAN, 2016). In addition to colonial impacts, contemporary race-based discrimination and practical barriers to care (e.g. rural residence) reduce the health of an already vulnerable population (Nelson and Wilson, 2018).

Indigenous and non-Indigenous health scholars have argued that reducing Indigenous health disparities requires partnerships between healthcare professionals and the communities they work with (Crowshoe et al., 2019; Henderson et al., 2018). For these partnerships to evolve, a healthcare workforce that is ready to practice (RTP) with these unique populations needs to be identified and/or trained (Crowshoe et al., 2019). An emerging body of literature exists attempting to define what it means to be RTP with distinct cultural groups. Daly et al. described RTP as “[concerning] the acquisition of clinical, professional and cultural skills required for successful practice” (Daly et al., 2013). Unfortunately, this definition has not been thoroughly investigated for Indigenous clients, which is a gap in the literature this study seeks to help fill. The lacking definition of RTP in Indigenous contexts hampers assessment, evaluation, and theorizing about important moderators of patient care delivery.

To date, a systematic review or analysis of what it takes for clinicians to be RTP with Indigenous peoples does not exist. This review will serve as the foundation for the future development and implementation of training and assessment tools to measure longitudinal changes in an individual’s RTP in Indigenous contexts. While some assessment tools exist, they are severely limited by their focus on specific clinical areas and reliance on subjective self-reporting (David et al., 2006). Such an approach requires a unilateral understanding of Indigenous RTP, which is not fully delineated in the current literature. The overarching aim of this review is to summarize what it means to be clinically RTP with Indigenous populations and how healthcare professionals can tailor their care to be ethical and effective when working with Indigenous patients.

## Methods

We used a quantitative content analysis (Krippendorf, 2004) to analyze peer-reviewed articles identified through a comprehensive search strategy, in alignment with the recommendation for literature-based content analyses (Gaur & Kumar, 2018) and the Preferred Reporting Items for Systematic Reviews and Meta-analyses (PRISMA) 2020 statement (Page et al., 2021). The original protocol of the study was registered with Open Science Framework (10.17605/OSF.IO/7SZWV). The combined systematic review and content analytic methods produced quantitative data regarding trends in the literature that could statistically derive domains that identify a clinician who is RTP with Indigenous populations across four countries where settler colonialism took place (i.e. Canada, the United States, New Zealand, and Australia).

### Search strategy

The search strategy was developed with research librarian KAH. MEDLINE ALL (Ovid), Embase (Ovid), APA PsycInfo (Ovid), CINAHL Plus with Full Text (Ebsco), Social Work Abstracts (Ebsco), and Web of Science Core Collection (Clarivate) were searched in January 2022. The search focused on two main concepts: Indigenous and readiness to practice. For each concept, both keywords and subject headings were searched. Keywords were the same across all databases, and subject headings were determined by the specific database’s indexing. The draft search in Medline was piloted against known relevant studies; as well, the team reviewed the search. Where required, revisions were made, and then the Medline search was translated for all other databases. The full search strategy for each database can be seen in Appendix 1, Table 2. References of relevant articles were hand searched.

### Study selection

Studies that explored the domains of RTP within an Indigenous context were included. This comprised any text discussing relevant skills or knowledge needed to be deemed fit to practice. There were no constraints on profession, and fields such as nursing, medicine, social work, education, and dentistry were all eligible. Articles were sourced from the CANZUS countries (i.e. Canada, Australia, New Zealand, and the USA), due to their similar historical and political relationships with Indigenous populations and current disparities across a range of metrics (Murry et al., 2022), including health (Smylie et al., 2010). Inclusion criteria included publication in the last 20 years, peer-review, and in the English language. Additionally, Indigenous peoples had to be the sole primary focus area of the article. Dissertations, commentary, and opinion pieces were excluded.

### Study retrieval and data extraction

Search results were uploaded to Covidence, and auto-deduplicated. The inter-rater calibration exercise took place before screening titles and abstracts to ensure all screeners are applying the inclusion and exclusion criteria correctly. Titles were screened independently by the first and third authors in Covidence. Disagreements were resolved by consensus and if consensus was not possible, disagreements were resolved through discussion with the second author. After the abstract screening, full-text studies were retrieved and then screened independently by the first and third authors. Again, disagreements were resolved by consensus or review by the second author. The reasons for exclusion at this full-text stage were documented.

### Search results

**Fig. 1:**
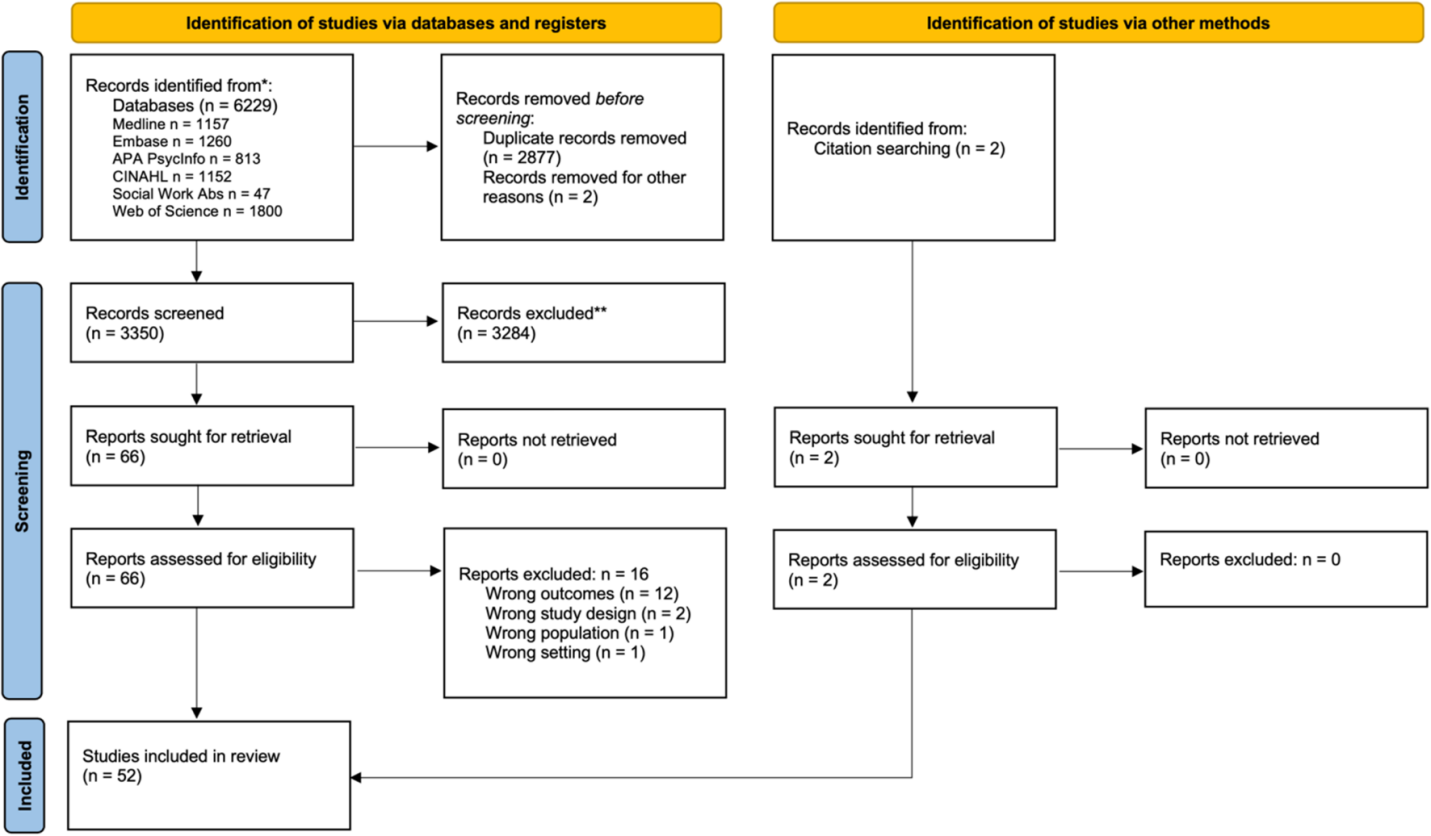
PRISMA flow diagram

After duplicates were removed, the database search retrieved 3350 records. Upon review and extraction, the final sample included 52 articles, which includes two additional studies identified through reference searching. The PRISMA flow diagram outlines reporting at all stages (see Figure 1). Inter-rater agreement for screening process was 85% according to percent agreement and 0.77 according to Cohen’s Kappa, indicating substantial agreement between both parties (Gisev et al., 2013).

### Coding procedure

The included studies (*n* = 52) were subjected to content analysis following the process outlined by Krippendorf (2004) and adapted for empirical literature (Gaur & Kumar, 2018). The process involves four stages: (1) data collection of a corpus of materials (described above); (2) coding those materials for attributes or constructs of interest; (3) analysis (described below); and (4) interpretation of coded content. To identify attributes of healthcare professionals who are RTP with Indigenous populations, we used an inductive coding procedure where codes were created as variables and constructs related to RTP appeared in the articles under review. Once a code was created, all articles were coded as either having the code’s variable or construct present or not, i.e. using a binary rating scale of 1 for present or 0 for absent. The process uncovered 56 topical codes (see Table 1 for the full list). Every article was independently coded by the first and third authors. Across the 52 articles and 56 codes, the coders agreed 91.86% of the time (Kappa = 0.83 [SE = 0.01], 95% CI = 0.82–0.86), or near-perfect agreement (Gisev et al., 2013). Ratings were averaged across the two coders.

**Table 1:** Readiness to practice items for Indigenous populations by principal component analysis component loading, variance explained, and internal agreement (i.e. Cronbach’s alpha)

Code	Relational disposition	Decolonized practice	Cultural immersion	Indigenous professional support
Providing support to family	−0.82			
Trustworthiness	−0.81			
Confidentiality	−0.80			
Creates welcoming spaces	−0.79			
Patient-perceived acceptability	−0.78			
Adaptability	−0.77			
Indigenous consultations about care	−0.72			
Clear effective communication	−0.72			
Patient-centred care	−0.70			
Authentic partnership	−0.69			
Positive attitude and personality	−0.65			
Reducing complex language	−0.53			
Rejection of pan-Indigenous concept	−0.52			
Making care accessible	−0.51			
Culturally considerate solutions	−0.49			
Emotional engagement	−0.46			
Considerate of cultural norms	−0.44			
Acknowledgement of disparities		0.74		
Deconstruction of prejudice		0.68		
Awareness of racism		0.68		
Awareness of cultural environment		0.67		
Developing empathy through immersion		0.63		
Goal of sustainable change		0.63		
Decolonized delivery		0.60		
Openness to tradition knowledge		0.59		
Integrates traditional knowledge		0.57		
Understands geographical influences		0.54		
Appreciates Indigeneity in modern contexts		0.49		
Understands history of colonization		0.48		
Works to be an ally		0.47		
Engages in ongoing learning		0.44		
Provides holistic care		0.41		
Immersion in local culture			−0.89	
Experiential learning			−0.87	
Immersion in local community			−0.83	
Centrality of context			−0.64	
Community-based participatory methods			−0.57	
Indigenous peer mentorship				−0.84
Accessible peer support				−0.77
Indigenous clinicians as role models				−0.77
Working with peers in small groups				−0.46
Practices to facilitate safe spaces				−0.43
Percent of total item variance explained by component:	21%	15%	10%	8%
Cronbach’s alpha of single-component loading items:	.91	.84	.85	.71

Item loadings below 0.40 not shown. Cross-loading and non-loading items not shown

### Analysis

Content analysis ratings were subjected to a principal components analysis (PCA) using varimax rotation on their polychoric correlation matrix. Polychoric correlations are the appropriate statistic when variables are ordinal or binary rather than continuous (O’Connor, 2022). Prior to running our PCA on the quantitatively coded RTP constructs, we inspected the KMO index to ensure there was enough shared variance between the codes to justify looking for underlying dimensions. The measure of sampling adequacy was 0.83, considered meritorious according to Kaiser and Rice’s (1974) standard. To determine the number of factors to estimate with our PCA, we then ran a Velicer’s (1976) MAP test, which iteratively runs PCAs to find the solution that explains the most amount of systematic variance. The MAP test showed that four components were the ideal solution. The KMO test, MAP test, and PCA were run using the *EFA.dimensions* package (O’Connor, 2022) in R version 4.2.1 (R Core Team, 2022).

## Results

The four extracted components, representing what the literature has identified that healthcare professionals need in order to be RTP in Indigenous populations (RTPI), were (1) a relational disposition, (2) decolonized practice, (3) cultural immersion, and (4) Indigenous professional support (see Table 1 and discussion below). The four-component solution explained 54% of the variance among the articles’ codes. Only items loading above 0.40 on a single factor were used for interpretation (Kline, 2016). The internal consistency of each factor was at or above the traditional cut-off of 0.70 (John & Benet-Martinez, 2000), at *ɑ* = 0.91, 0.84, 0.85, and 0.71, respectively. Appendix 2 includes a list of the 52 included studies for reference.

## Discussion

Our systematic review and subsequent content and factor analyses of the empirical literature regarding healthcare professionals RTPI identified four key components specific to Indigenous peoples. These components included (1) a relational disposition, (2) decolonized practice, (3) cultural immersion, and (4) Indigenous professional support. We discuss each in more detail below.

### Relational disposition

We named the first component of RTPI “relational disposition”. In order of component-loading strength, relational disposition items included “Providing Additional Family Support”, “Individual Trustworthiness”, “Confidentiality”, “Creation of Welcoming Spaces”, “Patient-perceived Acceptability”, “Adaptability [Open minded and flexible to different attitudes and personal schedules]”, “Indigenous Consultation in Decision Making”, “Clear and Effective Communication”, “Individual Centred Care [Patients actively involved in care planning with options given]”, “Authentic Partnership [non-performative relationships with Indigenous people that aim to be collaborative]”, “Positive Attitude and Personality”, “Reducing Complex Language Use”, “Rejection of Pan-Indigenous Concept”, “Continuity and Accessibility of Care”, “Culturally Considerate Healthcare Solutions”, “Emotional Engagement”, and “Acknowledgment of Cultural Norms”.

On the surface, this component of RTPI describes one’s conduct that is largely related to prosocial interpersonal behaviours, or personality characteristics associated with prosocial interpersonal behaviours. Actions such as being trustworthy, positive, or emotionally engaged, creating welcoming spaces, and protecting patients’ confidentiality all insinuate positive or polite interactional elements of the clinician-patient relationship. In alignment with this, items such as supporting the family’s involvement in the patient’s care, involving the patient in decision making, reducing complex language, trying to be clear and effective when communicating, practicing patient-centred care, authentically partnering with the patient, and making continuity of care accessible describe actions and behaviours that attempt to reduce status differences between clinician and patient. Further, this component contained several Indigenous-specific items, such as consulting with Indigenous care providers (e.g. midwives) in care delivery and infrastructure, rejecting pan-Indigenous perspectives that ignore diversity among and within Indigenous peoples, and being considerate of cultural behavioural norms (e.g. length of time between turn taking). We chose to label these mental processes “relational disposition” due to how these processes implicate meanings that are specific and significant to living out Indigenous worldviews and their consequent ethics.

By the use of the term relational in naming this construct (i.e. *relational disposition*), we invoke its association with the ontology of relationalism, as articulated by Wilson’s Research is Ceremony 2008 works. Relationalism is a view of reality shared among many Indigenous cultures, where what constitutes being, knowing, and value flows from an explicit recognition of our interdependence on proximal and distal relationships, in the past, present, and future, that are complex, in flux, holistic, cyclic, and natural, even in their metaphysics (Aikenhead & Ogawa, 2007; Cajete, 2000; Wilson, 2008). Consequently, humans should recognize that their position is temporary, not fixed, and equal to others, versus superior, due to its dependency on those relationships. While a full discussion of relationalism’s ontological, epistemological, and axiological implications is beyond the scope of this paper, e.g. comparing relationalism to post-positivism or social constructivism, the ethical implications are relevant.

Ethical behaviours as construed in Indigenous cultural worldviews have been described in the contexts of health promotion (e.g. McPhail-Bell et al., 2015), healthcare worker training (e.g. Parker et al., 2019), psychiatry (e.g. Brant, 1990), counselling (e.g. McCormick, 1998), social work (e.g. Burnette et al., 2014), research (e.g. Ball & Janyst, 2008), program evaluation (Julian et al., 2017), resource extraction (e.g. Lertzman & Vredenburg, 2005), and legal discourse (e.g. Ermine, 2007). Unsurprisingly, the ethical standards delineated across these fields significantly overlap, sometimes cross-pollinating one another. For example, the influential Four R’s ethical framework in higher education by Kirkness and Barnhardt (2001) has been adopted and adapted for literary studies (Brunette-Debassige & Wakeham, 2020), qualitative research methods (Snow, 2018), educational research (McGregor & Marker, 2018), grant preparation programs in science (Grant et al., 2022), school principal training programs (Tessaro et al., 2018), and global communications (Harris & Wasilewski, 2004). Importantly, like many of the other ethical commentaries available, the Four R’s framework of respect, relevance, reciprocity, and responsibility deductively flows from the ontological depictions of relationalism, where acting ethically means working to stay in good standing within the web of relationships in which one is entrenched. We see many of the items that loaded on this component as aligning with ethical principles or expressing behaviours associated with relationalism (e.g. holistic perspective of the patient and working to reduce the power of status).

By naming this component “disposition”, we use its definition as, “an attitude of mind, especially one that favors one alternative over others” (https://www.vocabulary.com/dictionary/disposition). In this sense, a clinician with a relational disposition is someone who prioritizes respectful relationships and considers patients’ lives in a wider context, rather than relating in a paternalistic fashion or considering patients as solely a presenter of symptoms. In our data, a relational disposition was composed of independent behaviours (e.g. confidentiality), interactions with the patient (e.g. reducing complex language), and attempts to understand patients’ cultural context (e.g. rejecting pan-Indianism^1^). It is important to note, however, that other behaviours not represented in our codes could also fulfill this aspect of the clinician’s RTP, such as relationship building with Indigenous patients and moving away from time-constrained and solely task-oriented outcomes (O’Connor et al., 2015). Interestingly, this component explained the most amount of variance among our codes, suggesting that it is the most important element of a clinician’s RTPI in terms of patient and practitioner perceptions depicted in the research.

### Decolonized practice

Decolonized practice was the second component of RTPI, explaining 15% of the items’ variance. It comprised the following codes in order of component loading strength: “Acknowledging Indigenous Health Disparities [Internalized need for positive change in Indigenous healthcare outcomes]”, “Prejudice Deconstruction”, “Racism and Discrimination Awareness”, “Culturally Appropriate Learning Environment”, “Developing Empathy Through Learning [Indigenous teachings]”, “Goal of Sustainable Change”, “Decolonized Healthcare Delivery”, “Open Mindedness to Traditional Knowledge”, “Integration of Traditional Knowledge”, “Recognition of Geographical Complexity”, “Modernity of Indigeneity”, “Deep Historical Understanding”, “Clinician Allyship and Advocacy”, “Importance of Ongoing Learning [long-term commitment to learning about Indigenous culture]”, and “Delivering Holistic Care”.

The codes comprising the decolonized practice domain focus on internalized understandings of Indigenous issues and actionable steps that can be taken to reduce colonial experiences of Indigenous peoples throughout care delivery processes. The component was heavily loaded with cognitive items, in terms of (1) knowledge, e.g. ongoing learning about current states (e.g. health disparities, Indigeneity in modern contexts) and historical influences (e.g. history of colonialism), and (2) awareness (e.g. of racism, cultural environment, and geographical influences). Actions associated with decolonized practice included clinicians unpacking how prejudice influences healthcare processes (i.e. deconstruction of prejudice), attempting to rid those processes of prejudice (i.e. decolonized delivery), being open to integrating traditional knowledge, and providing holistic care. Additionally, there were a few items that reflected longer-term active commitments (e.g. works to be an ally, goal of sustainable change) and affective states (e.g. empathy).

Decolonizing practices have been attempted and described in several fields of health and medicine. For example, attempts to decolonize practices have been made in medical school training (Henderson et al., 2023), cancer care (Beckett et al., 2021), medical decision-making (Cohen-Fournier et al., 2020), mental health (Lewis et al., 2018), and COVID response (Richardson & Crawford, 2020, see also Barnabe et al., 2022), while recommendations have been articulated for end-of-life care (Anderson & Woticky, 2018) and the therapeutic relationship (Dupuis-Rossi, 2021). Research has demonstrated the need for decolonizing practices at the first stage of diagnosis (Smith-Morris et al., 2021), diabetes care (Pilon et al., 2019), and the treatment of rheumatic heart disease (Haynes et al., 2021). All of these examples include objectives and recommendations similar to our second component of RTP, such as recognizing racism, knowing the history, and amending practices (e.g. Swidrovich, 2020). Researchers and clinicians in this space who are interpreting data using constructs adapted from the ontological considerations of Indigenous methodologies are one way that Indigenous theorizing can take place within predominantly Western spaces and decolonize research (Kovach, 2021), ideally for the benefit of both scientific understanding and the continuance of Indigenous Ways of Knowing and Being (e.g. Drew & Henne, 2006).

While this literature helps to corroborate our component on decolonizing practices, our findings suggest they are only a part of RTP. It is important to stress that decolonizing practices is not a short-term investment. In our data, there were sessions on how to decolonize practices that were delivered in short-term educational units. A few studies reported short-lived and sometimes negative impacts on the RTP of some participants (Bullen and Roberts, 2019). One possibility as to the reason for the negative effects of short-term educational interventions may be participants’ admitted pre-educational overconfidence in understanding Indigenous issues and their subsequent post-educational misunderstanding of Indigenous issue complexity (Wolfe et al., 2018). Another possibility for the limited effectiveness is the difficult nature of confronting personal limitations and the necessity of longer-form delivery methods (Bullen and Roberts, 2019). In short, a conflict exists between the need for long-term personal RTP development and the short-term delivery format of many decolonized practice interventions.

### Cultural immersion

The cultural immersion component, comprising 10% of the item variance, was formed from the following codes in order of component-loading strength: “Cultural Immersion [cultural awareness development]”, “Experiential Learning”, “Indigenous Community Immersion [community placements for clinical and/or learning experience]”, “Centrality of Context”, and “Community Based Participatory Research Approach”.

An overarching theme from the reviewed literature was the importance of trainees being physically present in Indigenous communities. Throughout the literature reviewed, trainees transitioning into full practice discussed the importance of having real-world experiences to accompany their Indigenous educational learnings (Malau-Aduli et al., 2022; Warren et al., 2016). One study concerning rural healthcare professionals working long-term in Indigenous communities discussed how those who worked and lived in Indigenous communities experienced a significant increase in self-perceived readiness to work within these contexts (Lindeman et al., 2014).

Being immersed in cultural life helps healthcare trainees contextualize lifestyle differences and gain appreciation for the difficulties that exist within the communities they were training to serve (Malau-Aduli et al., 2022; Thackrah et al., 2014). Trainees who work within these communities have been shown to be more likely to work within Indigenous contexts after graduation (Larson et al., 2011).

The benefit of cultural immersion is not a healthcare-specific notion, as many allied fields, including social work (Thibeault, 2019) and education (Burgess & Cavanagh, 2016), discuss the importance of understanding the cultural norms, beliefs, and practices of an Indigenous community. Experiential learning opportunities provide opportunities to interact with Indigenous people and gain exposure to their ways of knowing, doing, and being, the complexities these individuals face, and ways to improve care delivery for Indigenous patients.

### Indigenous professional support

The Indigenous professional support domain, representing 8% of the item variance, was formed from the following codes in order of component loading strength: “Indigenous Peer Mentorship”, “Accessible Peer Support [both Indigenous and non-Indigenous peer support]”, “Indigenous Clinicians as Role Models”, “Enhanced Learning via Small Groups [helps better internalize Indigenous knowledge and teachings]”, and “Safe Space Creation [where individuals feel safe to voice opinions and learn from mistakes]”.

A common trend within the literature surveyed was the admission of healthcare professionals’ desiring but having limited Indigenous professional peers supports when trying to navigate the commonly complicated cases presented by their Indigenous patients (Browne et al., 2013; Turner et al., 2014). The need for Indigenous professional presence was lacking throughout clinicians’ and allied healthcare professionals’ training as well as their practicing years. Respondents commented on the desire for Indigenous superiors as role models to aid in understanding Indigenous conceptual teachings and to provide a safe space to ask difficult questions (Huria et al., 2017). RTPI may be also positively affected by simply increasing the number of Indigenous healthcare professionals within various areas of healthcare as a means of enhancing Indigenous professional peer support capacity and representation (Rohatinsky et al., 2018).

## Implications and applications

Within the larger body of literature on non-Indigenous RTP, this study demonstrated that practicing medicine in Indigenous populations is a unique experience that medical school may not prepare one for. The requirements of being RTPI are different from RTP in the stage of one’s career when it is relevant and in its content. For example, non-Indigenous RTP is conceptualized as one’s professional maturation following the transition out of medical school and into practice. RTPI, on the other hand, becomes relevant whenever a practitioner encounters Indigenous patients for the first time regardless of where they are at in their career. In terms of content, RTP appears to mean something different depending on whether one is transitioning from school to practice or from a non-Indigenous to an Indigenous patient population. A recent content analytic review of non-Indigenous RTP literature (Le Huray et al., 2023) identified seven domains of RTP (e.g. clinical experience, social, and professional development experiences), or standards by which peers, supervisors, and the clinicians themselves judge their RTP. None of the RTP domains in Le Huray et al.’s analysis overlapped with our four components of RTPI. Together, these findings show that being RTP in one setting does not guarantee RTP across settings.

A second contribution of this study is consolidation of a growing body of literature about Indigenous healthcare specific to best practices for clinicians. Prior to this content analytic review, previous work on RTPI was scattered across disciplinary outlets (e.g. nursing, medicine, social work, education, and dentistry) and had not been summarized. Our four-part definition of RTPI adds both clarity and complexity to the construct. Many articles focused on what we identified as individual components of RTPI (e.g. decolonizing practices or immersion experiences in clinician training), while we were able to describe the attributes, practices, learning experiences, and supports needed to become ethically and effectively RTP with Indigenous peoples. Determining what underpins Indigenous RTP helps to inform clinicians about what constitutes ethical high-quality care for Indigenous patients and their communities.

Our four-part definition of RTP can be useful to healthcare practitioners, health science educators and students, and agencies looking to hire, assess, or train their employees. Healthcare practitioners who desire to be more effective or prepared to work with Indigenous patients can use our four-part definition as a starting point for self-development. Health science programs might consider how curriculum and/or learning experiences can be offered to help prepare students to be RTP. For healthcare agencies, our four-part definition of RTP might be converted into a scale and added as a selection measure to screen in or out applicants who are or are not RTPI. Incentive programs, performance evaluations, and workplace punishment policies could use RTPI as a basis for employee assessment and rewards for desired behaviour. Finally, RTPI could inform training content within hospitals, urgent cares, or health centres that regularly serve Indigenous patients. While our four-part definition of RTP has not been validated in terms of (e.g. correlations with) patient outcomes, it is the only quantitatively derived definition of RTP with Indigenous populations that exists. As a study of studies, our four-part definition represents the most empirically based set of recommendations available.

### Limitations and future research

As with any study, there are limits to what we can conclude with confidence based on our design and data. For one, while our data described programs attempting to improve clinicians’ RTPI and patients’ and practitioners’ reflections on what helped clinicians be RTPI, we did not have data correlating these components with patient outcomes. Future work should involve piloting these four areas, assessing the extent to which clinicians utilize them, and tracking patient outcomes such as patient satisfaction, compliance, or health empowerment metrics, to see whether RTPI produces the impacts we expect. It is possible that there are other factors at play beyond RTPI that help or hinder patient benefits. Predictive analyses should also consider moderators (e.g. adequate hospital resources, staffing) and mediators (e.g. hours of training or experiential learning) of the proposed relationship between RTPI and improved healthcare experiences.

To collect a sample of literature sizable enough to draw conclusions, we sought out studies across four nations with similar relationships between Indigenous peoples and the nation-state due to shared British, settler colonial histories. While this improves the stability of our estimates, it also means our definition of RTPI aggregates across international differences. To ensure that our findings are reflective of Indigenous peoples within Canada, for example, the four-part definition should be shared with an expert panel or town hall of Indigenous community members in the location where training is to take place. Local perspectives could confirm, extend, or reject components of our definition, which could help tailor these results to the immediate context.

## Conclusion

The need for healthcare practitioners who are equipped to practice in Indigenous populations is clear. Persistent disparities, cultural differences, experiences with colonialism, and national commitments to reconciliation highlight the gaps in typical healthcare education and services and the needs of Indigenous patients. Our review identified dozens of primary studies documenting clinician knowledge, skills, behaviours, awareness, and dispositions believed to be effective in Indigenous healthcare. Our analyses quantitatively reduced the recommendations from these disparate studies to identify four underlying components of RTP with Indigenous populations: a relational disposition, decolonized practice, cultural immersion, and support from Indigenous professionals. The four-part definition provides a framework for self-assessment, curriculum design, and workplace performance assessments aimed at promoting and improving RTPI. It is our hope that this study will help move the conversation beyond what Indigenous peoples need from their physicians and nurses, to how their physicians and nurses can become RTPI as part of their standard training. The more practitioners are RTPI, the more doctor-patient interactions will be culturally safe and likely to result in the intended benefits of care. With time, perhaps improving RTPI will help address current health disparities and remedy the colonial history that bore them.

## Contributions to knowledge

What does this study add to existing knowledge?

- Offers an empirically derived definition of what is required of healthcare practitioners to be ready to practice with Indigenous populations.
- Provides a succinct analysis of heterogenous literature regarding what makes an individual prepared to practice with Indigenous patients.
- Summarizes repeated and mostly qualitative articulations of healthcare practitioners’ best practices using advanced statistical techniques.
- Moves research from exploratory to confirmatory stages.

What are the key implications for public health interventions, practice, or policy?

- To mitigate ongoing healthcare disparities for Indigenous peoples in Canada, it is crucial to provide healthcare professionals with culturally appropriate training and education for effectively working with Indigenous patients, a pivotal step in narrowing health outcome disparities among Indigenous patients.
- This study provided evidence for four concrete areas for potential investment in training and assessment. Individuals and programs can be evaluated on how well they fulfill each quadrant.
- Improving the quality of Indigenous healthcare can positively contribute to overall public health of Canada and potentially reduce rates of chronic disease and cost to the healthcare system.

## Data Availability

The data that support the findings of this study are available from the corresponding author upon request.

## Appendix 1

Search strategy

**Table 2:** Database(s): **Ovid MEDLINE(R) and Epub Ahead of Print, In-Process, In-Data-Review & Other Non-Indexed Citations and Daily**

**#**	**Searches**	**Results**
1	american native continental ancestry group/ or indians, north american/ or alaskan natives/ or indigenous canadians/ or american natives/ or indigenous peoples/	16,580
2	Oceanic Ancestry Group/	11,364
3	Health Services, Indigenous/	3737
4	(Aboriginal or First Nation* or Indigenous or Inuit* or Metis or eskimo* or Pacific Islander* or Torres Strait Islander*).tw,kf.	55,735
5	(native* adj2 (Alaska* or Hawaiian* or American* or Canadian* or Australian*)).tw,kf.	11,802
6	("American Indian*" or Amerindian*).tw,kf.	9574
7	(Native* adj2 (man or men or women or woman or boy* or girl* or child* or adolescent* or youth or youths or person* or adult* or people* or student* or Indian or Indians or Nation or Nations or tribe* or tribal or band or bands or community or communities or group or groups or population* or patient*)).tw,kf.	13,979
8	(tribal adj2 (people* or communities or community or resident* or group* or urban or person* or nation* or setting*)).tw,kf.	1420
9	or/1-8	90,035
10	Cultural Competency/	6189
11	(Cultural* adj2 (competen* or care or knowledge or skill* or awareness or capacit* or practic* or preparat* or appropriate or safety or capabilit* or placement*)).tw,kf.	17,095
12	(transcultur* or trans-cultur*).tw,kf.	2945
13	or/10-12	22,990
14	Curriculum/	81,718
15	education/ or education, professional/	24,450
16	exp Education, Medical/	176,929
17	exp Education, Nursing/	87,017
18	(Curriculum or curricula or education* or pedagog* or teach* or syllabus or course).tw,kf.	1,374,630
19	or/14-18	1,517,870
20	9 and 13 and 19	943
21	((prepare or prepared or preparing or preparedness or preparation or under-prepar* or underprepar* or un-prepar* or unprepar* or well-prepar*) adj10 (work* or care or caring or practic* or serve* or service*)).tw,kf.	44,540
22	((ready or readiness) adj10 (work* or care or caring or practic* or serve* or service*)).tw,kf.	7319
23	(practice adj3 model*).tw,kf.	7492
24	or/21-23	58,431
25	9 and 24	366
26	20 or 25	1273
27	limit 26 to yr="2001 -Current"	1142

## Appendix 2

**Empirical studies included for content analysis**

Abuzar, M. A., & Owen, J. (2016). A community engaged dental curriculum: A rural Indigenous outplacement programme. Journal of public health research, 5(1), jphr-2016.

Barnabe, C., Kherani, R. B., Appleton, T., Umaefulam, V., Henderson, R., & Crowshoe, L. (2021). Participant-reported effect of an Indigenous health continuing professional development initiative for specialists. BMC Medical Education, 21, 1-8.

Browne, J., Thorpe, S., Tunny, N., Adams, K., & Palermo, C. (2013). A qualitative evaluation of a mentoring program for Aboriginal health workers and allied health professionals. Australian and New Zealand Journal of Public Health, 37(5), 457-462.

Bullen, J., & Roberts, L. (2019). Transformative learning: A precursor to preparing health science students to work in Indigenous health settings?. The Australian Journal of Indigenous Education, 48(2), 129-140.

Bullen, J., Roberts, L., & Hoffman, J. (2017). What predicts health students’ self-reported preparedness to work in Indigenous health settings?. The Australian Educational Researcher, 44, 71-87.

Chakanyuka, C., Bacsu, J. D. R., DesRoches, A., Dame, J., Carrier, L., Symenuk, P., ... & Bearskin, L. B. (2022). Indigenous-specific cultural safety within health and dementia care: A scoping review of reviews. Social Science & Medicine, 293, 114658.

Durey, A., Halkett, G., Berg, M., Lester, L., & Kickett, M. (2017). Does one workshop on respecting cultural differences increase health professionals’ confidence to improve the care of Australian Aboriginal patients with cancer? An evaluation. BMC Health Services Research, 17(1), 1-13.

Forsyth, C., Irving, M., Short, S., Tennant, M., & Gilroy, J. (2019). Strengthening Indigenous cultural competence in dentistry and oral health education: Academic perspectives. European Journal of Dental Education, 23(1), e37-e44.

Freeman, T., Edwards, T., Baum, F., Lawless, A., Jolley, G., Javanparast, S., & Francis, T. (2014). Cultural respect strategies in Australian Aboriginal primary health care services: Beyond education and training of practitioners. Australian and New Zealand Journal of Public Health, 38(4), 355-361.

Gerlach, A. J., Browne, A. J., & Greenwood, M. (2017). Engaging Indigenous families in a community‐based Indigenous early childhood programme in British Columbia, Canada: A cultural safety perspective. Health & Social Care in the Community, 25(6), 1763-1773.

Giordano, A. L., Prosek, E. A., Schmit, M. K., & Wester, K. L. (2020). “We are still here”: Learning from Native American perspectives. Journal of Counseling & Development, 98(2), 159-171.

Goforth, A. N., Nichols, L. M., Sun, J., Violante, A., Christopher, K., & Graham, N. (2022). Incorporating the indigenous evaluation framework for culturally responsive community engagement. Psychology in the Schools, 59(10), 1984-2004.

Haozous, E. A., & Neher, C. (2015). Best practices for effective clinical partnerships with Indigenous populations of North America (American Indian, Alaska native, first nations, Métis, and Inuit). Nursing Clinics, 50(3), 499-508.

Harms, L., Middleton, J., Whyte, J., Anderson, I., Clarke, A., Sloan, J., ... & Smith, M. (2011). Social work with Aboriginal clients: Perspectives on educational preparation and practice. Australian Social Work, 64(2), 156-168.

Harvey, P., Nightingale, C., & Kippen, R. (2021). Rural medical students' self-reported perceptions of preparedness to practice in the Aboriginal and Torres Strait Islander Health Context. Australian Journal of Rural Health, 29(2), 261-266.

Hayward, M. N., Mequanint, S., Paquette-Warren, J., Bailie, R., Chirila, A., Dyck, R., ... & Harris, S. (2017). The FORGE AHEAD clinical readiness consultation tool: A validated tool to assess clinical readiness for chronic disease care mobilization in Canada’s first nations. BMC health services research, 17, 1-13.

Hill, B., Tulloch, M., Mlcek, S., & Lewis, M. (2020). The ‘within’ journey: Assessment of the online Indigenous Australian cultural competence training programme at Charles Sturt University. The Australian Journal of Indigenous Education, 49(1), 14-22.

Huria, T., Palmer, S., Beckert, L., Lacey, C., & Pitama, S. (2017). Indigenous health: Designing a clinical orientation program valued by learners. BMC Medical Education, 17(1), 1-8.

Jackson, D., Power, T., Sherwood, J., & Geia, L. (2013). Amazingly resilient Indigenous people! Using transformative learning to facilitate positive student engagement with sensitive material. Contemporary nurse, 46(1), 105-112.

Jumper-Reeves, L., Dustman, P. A., Harthun, M. L., Kulis, S., & Brown, E. F. (2014). American Indian cultures: How CBPR illuminated intertribal cultural elements fundamental to an adaptation effort. Prevention Science, 15, 547-556.

Kamaka, M. L., Paloma, D. S., & Maskarinec, G. G. (2011). Recommendations for medical training: A Native Hawaiian patient perspective. Hawaii Medical Journal, 70(11 Suppl 2), 20.

Kerrigan, V., Lewis, N., Cass, A., Hefler, M., & Ralph, A. P. (2020). “How can I do more?” Cultural awareness training for hospital-based healthcare providers working with high Aboriginal caseload. BMC Medical Education, 20, 1-11.

Kurtz, D. L. M., Janke, R., Vinek, J., Wells, T., Hutchinson, P., & Froste, A. (2018). Health sciences cultural safety education in Australia, Canada, New Zealand, and the United States: A literature review. International journal of medical education, 9, 271.

Lacey, C., Huria, T., Beckert, L., Gilles, M., & Pitama, S. (2011). The Hui Process: A framework to enhance the doctor-patient relationship with Maori. The New Zealand Medical Journal (Online), 124(1347).

Lewis, M., & Prunuske, A. (2017). The development of an indigenous health curriculum for medical students. Academic Medicine, 92(5), 641.

Lindeman, M., Dingwall, K., & Bell, D. (2014). Towards better preparation and support for health and social care practitioners conducting specialised assessments in remote Indigenous contexts. Australian Journal of Social Issues, 49(4), 445-465.

Macklin, C. D., Marchand, C., Mitchell, E., Price, R., Mitchell, V., & Bryant, L. (2021). Planting the seeds: Insights for researchers interested in working with Indigenous peoples. International Journal of Indigenous Health, 16(1).

Mahara, M. S., Duncan, S. M., Whyte, N., & Brown, J. (2011). It takes a community to raise a nurse: Educating for culturally safe practice with Aboriginal peoples. International Journal of Nursing Education Scholarship, 8(1).

Mattingly, J. A. (2021). Fostering cultural safety in nursing education: Experiential learning on an American Indian reservation. Contemporary Nurse, 57(5), 370-378.

Mauiliu, M., Sopoaga, F., & Ekeroma, A. (2013). Community experience of a Pacific Immersion Programme for medical students in New Zealand. The New Zealand medical journal (Online), 126(1376).

Moderie, C., Drouin, É., Rioux, R., Thommeret-Carrière, A. S., Béland, S., & Leduc, J. M. (2021). The Mini-Med School and Its Impact on Future Health Care Professionals' Attitudes toward Indigenous People. Journal of Health Care for the Poor and Underserved, 32(4), 2043-2054.

O’Connor, J., Chur‐Hansen, A., & Turnbull, D. (2015). Professional skills and personal characteristics for psychologists working in an urban Australian context with Indigenous clients. Australian Psychologist, 50(6), 464-474.

Oosman, S., Durocher, L., Roy, T. J., Nazarali, J., Potter, J., Schroeder, L., ... & Abonyi, S. (2019). Essential elements for advancing cultural humility through a community-based physical therapy practicum in a Métis community. Physiotherapy Canada, 71(2), 146-157.

Prodan-Bhalla, N., Middagh, D., Jinkerson-Brass, S., Ziabakhsh, S., Pederson, A., & King, C. (2017). Embracing Our “Otherness” A Mutually Transformative Journey in Delivering an Indigenous Heart Health Promotion Project. Journal of Holistic Nursing, 35(1), 44-52.

Purtzer, M. A., & Thomas, J. J. (2021). What Native Americans want nurses to know: Attitudes and behaviors desired in client/nurse relationships. Public Health Nursing, 38(2), 176-185.

Rohatinsky, N., Exner-Pirot, H., Parent-Bergeron, M., Bosevski, K., & Pratt, C. (2018). Indigenous Nursing Students’ Readiness for Practice Perceptions-Les perceptions d’étudiantes autochtones en sciences infirmières quant à leur préparation à la pratique. Quality Advancement in Nursing Education-Avancées en formation infirmière, 4(2), 3.

Sargeant, S., Smith, J. D., & Springer, S. (2016). Enhancing cultural awareness education for undergraduate medical students: Initial findings from a unique cultural immersion activity. Australasian medical journal, 9(7), 224-230.

Smith, K., Fatima, Y., & Knight, S. (2017). Are primary healthcare services culturally appropriate for Aboriginal people? Findings from a remote community. Australian journal of primary health, 23(3), 236-242.

Svarc, R., Davis, C., McDonald, H., Perruzza, J., Browne, J., Delbridge, R., ... & Palermo, C. (2018). Exploring the impact of Aboriginal health placement experiences on the preparation of dietetic graduates for practice with Aboriginal communities. Nutrition & Dietetics, 75(5), 448-456.

Thackrah, R. D., Thompson, S. C., & Durey, A. (2014). “Listening to the silence quietly”: Investigating the value of cultural immersion and remote experiential learning in preparing midwifery students for clinical practice. BMC Research Notes, 7, 1-12.

Thackrah, R. D., Thompson, S. C., & Durey, A. (2015). Exploring undergraduate midwifery students’ readiness to deliver culturally secure care for pregnant and birthing Aboriginal women. BMC Medical Education, 15(1), 1-9.

Thackrah, R. D., Wood, J., & Thompson, S. C. (2020). Cultural respect in midwifery service provision for Aboriginal women: Longitudinal follow-up reveals the enduring legacy of targeted program initiatives. International journal for equity in health, 19, 1-11.

Turner, K. M., Sanders, M. R., & Hodge, L. (2014). Issues in professional training to implement evidence‐based parenting programs: The preferences of Indigenous practitioners. Australian Psychologist, 49(6), 384-394.

Walton, J. (2011). Can a one-hour presentation make an impact on cultural awareness? Nephrology Nursing Journal, 38(1).

Warren, J. M., Irish, G. L., Purbrick, B., Li, J. J., Li, X., Fitzpatrick, D. J., & Faull, R. J. (2016). Developing the future Indigenous health workforce: The feasibility and impact of a student‐led placement programme in remote Indigenous communities. Australian Journal of Rural Health, 24(5), 306-311.

West, M., Sadler, S., Hawke, F., Munteanu, S. E., & Chuter, V. (2021). Effect of a culturally safe student placement on students’ understanding of, and confidence with, providing culturally safe podiatry care. Journal of Foot and Ankle Research, 14, 1-8.

West, R., Armao, J. E., Creedy, D. K., Saunders, V., & Rowe Minnis, F. (2021). Measuring effectiveness of cultural safety education in First Peoples health in university and health service settings. Contemporary Nurse, 57(5), 356-369.

West, R., Mills, K., Rowland, D., & Creedy, D. K. (2018). Validation of the first peoples cultural capability measurement tool with undergraduate health students: A descriptive cohort study. Nurse Education Today, 64, 166-171.

Woolley, T., Sivamalai, S., Ross, S., Duffy, G., & Miller, A. (2013). Indigenous perspectives on the desired attributes of medical graduates practising in remote communities: A Northwest Queensland pilot study. Australian Journal of Rural Health, 21(2), 90-96.

Wylie, L., McConkey, S., & Corrado, A. M. (2021). It’s a journey not a check box: Indigenous cultural safety from training to transformation. International Journal of Indigenous Health, 16(1).

Zhou, A. W., Boshart, S., Seelisch, J., Eshaghian, R., McLeod, R., Nisker, J., ... & Howard, J. M. (2012). Efficacy of a 3-hour Aboriginal health teaching in the medical curriculum: Are we changing student knowledge and attitudes?. Health Education Journal, 71(2), 180-188.

## Footnotes

1 By “pan-Indianism,” we refer to the generalization that “all Indians [Indigenous Peoples] are the same” and not the grassroots attempts for intertribal unity (Hertzberg, 1971). While there are similarities in relationship to the land and colonialism, it is an inaccurate and antiquated perception that the members of the hundreds of Nations lumped into the term “Indigenous” or “First Nations” or “American Indian” represent a monolithic group. This definition does not address the appropriateness of the term “Indian” which has known negative connotations rooted in colonialism that is not fully delineated in this text.

## Code availability

Available from the corresponding author upon reasonable request.
